# Aquatic beetles influence colonization of disparate taxa in small lentic systems

**DOI:** 10.1002/ece3.6845

**Published:** 2020-10-15

**Authors:** Matthew R. Pintar, William J. Resetarits

**Affiliations:** ^1^ Department of Biology and Centers for Water and Wetland Resources, and Biodiversity and Conservation Research University of Mississippi University MS USA; ^2^ Present address: Institute of Environment Florida International University Miami FL USA

**Keywords:** Coleoptera, community assembly, habitat selection, oviposition, priority effects, temporary ponds

## Abstract

Structure of natural communities is shaped by both abiotic characteristics and the ongoing processes of community assembly. Important to this process are the habitat selection behaviors and subsequent survival of colonists, both in the context of temporal changes in the abiotic characteristics and priority effects driven by earlier colonists. Aquatic beetles are prevalent in temporary freshwater systems, form speciose assemblages, and are often early colonists of temporary ponds. While beetles have the potential to influence community structure through post‐colonization interactions (predation and competition), our goal was to determine whether the presence of beetle assemblages (versus patches without beetles) influences the colonization and oviposition of a diverse group of animals in a naturally colonized experimental landscape. We established mesocosms that either contained existing beetle assemblages or contained no beetles and assessed abundances of subsequent colonists. Treefrogs, *Hyla chrysoscelis*, and mosquitoes, *Culex restuans*, both deposited fewer eggs in patches containing beetle assemblages, while two beetles, *Copelatus glyphicus* and *Paracymus*, colonized those patches at lower rates. One beetle, *Helophorus linearis*, colonized patches containing beetle assemblages at higher rates, while two beetles, *Berosus infuscatus* and *Tropisternus lateralis*, exhibited no colonization differences between treatments. Overall, there were no differences in the assemblage structure or richness of beetles that colonized patches. Our results illustrate the importance of species‐specific habitat selection behavior in determining the species composition of habitat patches, while emphasizing the role of priority effects in influencing patterns of community assembly. Habitat selection in response to abiotic and biotic characteristics of habitat patches can potentially create greater spatiotemporal niche separation among the numerous, often closely related species (phylogenetically and trophically), that can be simultaneously found in similar patches across landscapes.

## Graphical abstract

Aquatic beetles are prevalent in many lentic freshwater habitats. We experimentally tested if their presence can affect colonization by other organisms. We found aquatic beetles reduced oviposition by mosquitoes and treefrogs, with a mix of species‐specific effects on adult beetles.

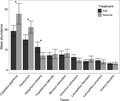

## 1 INTRODUCTION

Landscapes are mosaics of habitat patches that vary spatially and temporally in numerous abiotic and biotic characteristics (Hansson et al., 1995; Turner, 1989), and the processes that generate the distributions of organisms across landscapes are of fundamental interest in ecology (Chesson, 2000). Species composition of a habitat patch can be affected by abiotic patch characteristics, while species composition (both abundance and diversity) is itself a characteristic that becomes an integral component of future community assembly. Animals are expected to select and occupy patches based on maximizing their perceived expected fitness (Fretwell & Lucas, 1969), and competition, predation, resource availability, and abiotic habitat characteristics can all affect fitness and play roles in determining patch occupancy (Morris, 2003; Resetarits, 1996; Wiens, 1976).

In freshwater systems, the division between permanent and temporary is one of the most dominant abiotic characteristics influencing community structure, with fish being present in many permanent systems (Wellborn et al., 1996; Wilbur, 1997). In general, fish are effective predators of many other freshwater taxa, while potential prey, especially many aquatic insects and amphibians, have higher abundances and species richness in temporary, fishless habitats (Schneider & Frost, 1996). These distributions are due not only to direct consumptive effects of predators, but also to changes in prey behaviors (Lima, 1998; Orrock et al., 2008). In particular, habitat selection can have strong effects on species distributions: Prey attempt to avoid patches containing predators, whereas predators select patches containing more prey (Abrams, 2007; Höner et al., 2005; Lima, 2002; Peckarsky & Dodson, 1980; Pintar & Resetarits, 2017a; Resetarits & Wilbur, 1989; Veit et al., 1993). During the colonization process, adult aquatic insects and ovipositing amphibians select patches based on an array of patch characteristics, including predator (particularly fish) presence, patch size, and resource availability, among others (Pintar & Resetarits, 2017b; Resetarits et al., 2019; Trekels & Vanschoenwinkel, 2019; Vonesh et al., 2009). However, due to the dominant effects of fish (Pintar et al., 2018; Rieger et al., 2004), many insects and amphibians co‐occur in the same temporary fishless habitat patches (Wellborn et al., 1996; Wilbur, 1997).

In temporary pond communities, the order of arrival is an important consideration, as early‐arriving species can affect community dynamics through priority effects (Alford & Wilbur, 1985; Wilbur & Alford, 1985). In the southeastern United States, *Hyla chrysoscelis* (Cope's gray treefrog) is a common species that quickly and preferentially oviposits in newly filled temporary ponds (Pintar & Resetarits, 2017c), and *Culex restuans* is a generalist wetland mosquito that oviposits in similar habitats (Darsie & Ward, 2005). Similarly, adult aquatic beetles are able to quickly colonize and oviposit in these same habitats and form dense, speciose assemblages (Fairchild et al., 2000, 2003). Whereas larval *H. chrysoscelis* are herbivores and larval *Culex* are bacterivores, adult aquatic beetles can be predators, scavengers, or herbivores (Merritt et al., 2008), creating competition for treefrog larvae and other adult beetles, and predation pressure for treefrog larvae, mosquito larvae, and beetle larvae. For all of these taxa, habitat selection decisions can be critical as subsequent dispersal can either be unlikely (adult insects can lose wing muscles; Zera & Denno, 1997) or impossible until after metamorphosis (larval treefrogs, mosquitoes, and beetles). Given this potential for predation and competition, as well as their prevalence in many freshwater systems, we might expect the presence of beetles to affect colonization by other taxa.

We conducted a field mesocosm experiment using a naturally colonized experimental landscape to assess the effects of the presence of adult beetles on the subsequent colonization and oviposition behaviors of a variety of taxa (beetles, mosquitoes, treefrogs). For ovipositing taxa (mosquitoes and treefrogs) we predicted they would avoid patches containing beetles to both reduce competition and avoid predation, as they do in response to many other predatory taxa (Eitam & Blaustein, 2004; Pintar et al., 2018; Resetarits & Wilbur, 1989; Vonesh et al., 2009). For colonizing adult beetles, we did not have species‐specific predictions because (a) colonizing beetles often respond to variation in patch characteristics in species‐specific ways that can be unpredictable, even among closely related species (e.g., Pintar et al., 2018; Resetarits & Pintar, 2016; Resetarits et al., 2019), and (b) high densities of adult beetles are often found in natural habitats (Fairchild et al., 2000). Therefore, we could expect three outcomes for adult beetles in response to patches containing beetles: (1) avoidance to reduce the risk of predation or competition on themselves or their offspring, (2) no preference since high densities of beetles across landscapes may not present reliable differences among patches for selection to occur, and (3) attraction, as high insect densities may indicate high‐quality patches or the presence of potential mates (Sebastián‐González et al., 2010; Stamps, 1988).

## 2 MATERIAL AND METHODS

Our objective was to determine whether the presence of beetles affected colonization by adult beetles, and oviposition by treefrogs and mosquitoes. We assessed the responses of colonists to aggregate groups of beetles rather than specific beetle species because (a) the taxa used were those that naturally colonized our mesocosms, being representative of those dispersing across the landscape during the time of the experiment, (b) the presence and abundance of many of our beetle species are only marginally predictable in space and time, and (c) assessing the responses to numerous individual species would be difficult to control and greatly increase the size and scope of this experiment. Thus, we had two treatments: one in which beetles were added to mesocosms (Add) and one in which beetles were removed from mesocosms (Remove), effectively creating an experimental landscape where patches either contained a preexisting beetle assemblage or did not contain beetles, respectively.

**FIGURE 1:**
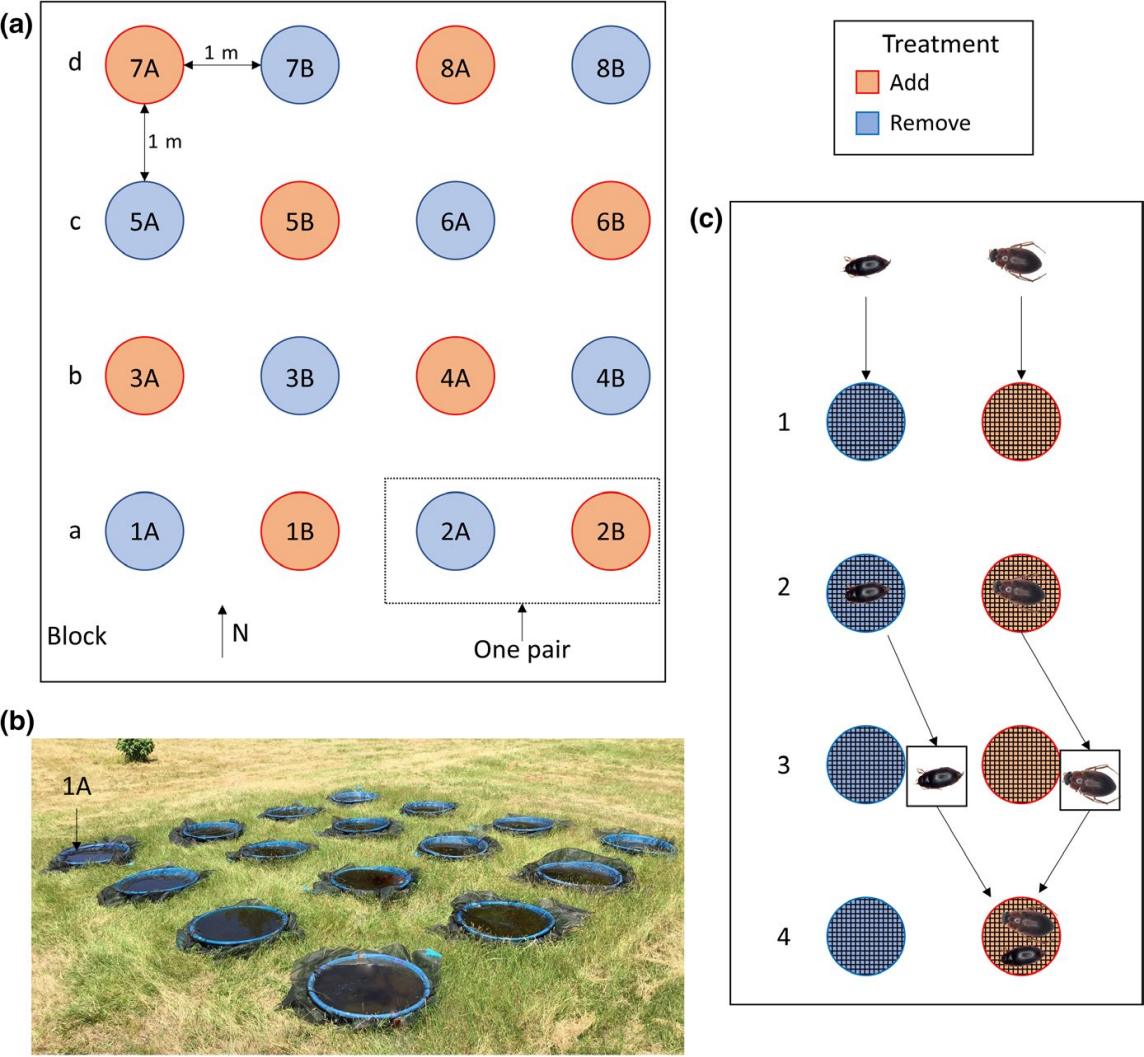
(a) Diagram (to scale) illustrating the experimental layout of the 16 mesocosms (1 m diameter) with pairs of mesocosms numbered 1–8, blocks (rows) lettered a–d, and colors representing treatment (Add or Remove). (b) Photograph of the array in May with mesocosm 1A labeled for reference. (c) Conceptual illustration of how treatments were established. Step 1: Insects colonize mesocosms above the screens. Step 2: Insects are collected from above the screens. Step 3: Insects are identified. Step 4: Insects are returned to mesocosms below the screens.

We established mesocosms (110 L plastic wading pools; 1 m diameter) on 17 April 2017 in a 4 × 4 array (16 total mesocosms; Figure 1) in a field with open canopy at the University of Mississippi Field Station (UMFS) in Lafayette County, Mississippi, USA. Mesocosms were separated by 1 m (edge‐to‐edge), filled with unchlorinated well water, and contained 0.25 kg of hardwood leaf litter (primarily Fagaceae) as a resource base to support primary and secondary productivity. Mesocosms were covered with tight‐fitting window screening (1.3 × 1.13 mm opening) that was depressed below the water surface to separate colonists from the leaf litter and prevent the escape of beetles that were below the screens within the mesocosms (Figure 1b). Beetles placed below the screens (see below) were still able to access the water surface along mesocosm margins. We allowed colonization to occur immediately after filling on 17 April. We counted and removed all *Culex* egg rafts oviposited in mesocosms on a daily basis. Frog eggs oviposited in mesocosms were collected daily, photographed, and returned to nearby fishless ponds. The total number of frog eggs laid in each mesocosm on each day was later counted from photographs using ImageJ (Bohenek & Resetarits, 2017; Schneider et al., 2012).

### 2.1 Insect processing and establishment of treatments

We allowed colonization by beetles to occur uninterrupted until 22 April. Beginning on 22 April, we exhaustively collected all colonizing beetles and other taxa from each mesocosm (above the screens) with fine mesh nets. From these samples, we removed, preserved, and later identified all small beetles that could potentially fit through the screen gaps (species with widths <1.5 mm). Non‐beetle insect taxa were removed from the mesocosms during collections and excluded from the study due to low abundances. All remaining beetles were identified to the lowest taxonomic level possible without preservation (see Table 1 for taxa). Identifications followed Pintar and Resetarits (2020), which were primarily based on Larson et al. (2000) and Epler (2010). These beetles were returned to mesocosms and placed below the tight‐fitting screens to separate them from future colonists and prevent exchange of individuals between above and below the screens (Figure 1c). We formed pairs of two adjacent mesocosms to create our two treatments (8 replicates each): Beetles from both mesocosms were aggregated and placed below the screen of one mesocosm (Add), while the other mesocosm did not receive any beetles below the screen (Remove). The treatment of the first mesocosm in the first block was randomly assigned, and then systematically alternated between mesocosms by row and column; no mesocosms of the same treatment were adjacent (Figure 1a). We continued this process of collecting, identifying, and placing beetles below the screens of mesocosms every other day until 9 June 2017, when the experiment was terminated. All insects collected above the screens were alive at the time of collection, and all beetles returned below the screens were alive at the time they were returned.

**TABLE 1:** Larger beetles (width >1.5 mm) that were placed below the screens of mesocosms

Family	Identified level	Most likely species	Abundance		
			During	Analyzed	At end
Dytiscidae	*Acilius mediatus*		1	1	0
Hydrophilidae	*Berosus*	*infuscatus* ^a^	130	127	50 (*infuscatus*)
Dytiscidae	*Celina*	*angustata, hubbelli*	3	3	0
Dytiscidae	*Coelambus nubilus*		1	1	0
Dytiscidae	*Copelatus chevrolati*		1	1	0
Dytiscidae	*Copelatus glyphicus*		396	376	120
Hydrophilidae	*Cymbiodyta chamberlaini*		9	9	0
Hydrophilidae	*Enochrus* sp. 1	*cinctus, consors, consortus*	4	4	0
Hydrophilidae	*Enochrus* sp. 2	*ochraceus* ^b^	92	89	4 (*ochraceus*)
Hydrophilidae	*Enochrus* sp. 3	*fimbriatus, hamiltoni, interruptus*	4	4	1 (*hamiltoni*)
Hydrophilidae	*Helochares maculicollis*		11	11	0
Helophoridae	*Helophorus*	*linearis* ^c^	157	148	0
Dytiscidae	*Hydaticus bimarginatus*		8	8	5
Noteridae	*Hydrocanthus*	*atripennis*	5	5	4 (*atripennis*)
Hydrochidae	*Hydrochus rugosus*		4	4	0
Dytiscidae	*Hydrocolus deflatus*		7	1	0
Dytiscidae	*Hydrocolus*	*oblitus* ^d^	5	5	0
Dytiscidae	*Hydroporus*	*rufilabris* ^e^	39	29	29 (*rufilabris* *)*
Dytiscidae	*Ilybius gagates*		2	2	2
Dytiscidae	*Laccophilus fasciatus*		78	78	3
Dytiscidae	*Laccophilus proximus*		70	70	33
Dytiscidae	*Meridiorhantus calidus*		1	1	1
Dytiscidae	*Neoporus blanchardi*		4	0	2
Dytiscidae	*Neoporus undulatus*		6	4	0
Haliplidae	*Peltodytes sexmaculatus*		1	0	0
Dytiscidae	*Platambus flavovittatus*		1	1	1
Dytiscidae	*Thermonectus basillaris*		5	4	3
Hydrophilidae	*Tropisternus blatchleyi*		5	5	1
Hydrophilidae	*Tropisternus collaris*		28	24	16
Hydrophilidae	*Tropisternus lateralis*		124	121	57
Hydrophilidae	*Tropisternus natator*		1	1	1

Note

The “During” column lists the abundance of that taxon identified in the field during the experiment, while the “At end” lists the abundances of that taxon collected from below the screens at the end of the experiment. The “Analyzed” column lists the number of individuals of those in the “During” column that colonized mesocosms after the collections on 22 April. For species with uncertainty (“Most likely species” column), we include the species‐level identifications of individuals collected at the end of the experiment in parentheses (all individuals in each row at the end were of the same species).

^a^ *Berosus sayi* is another large *Berosus* that regularly occurs at UMFS, but all identified individuals were *B. infuscatus*, and >90% of these two species at UMFS are *B. infuscatus* (Pintar & Resetarits, 2017b).

^b^ *Enochrus blachleyi, E. pygmaeus,* and *E. sayi* all regularly occur at UMFS, but *E. ochraceus* is by far the most common.

^c^ *Helophorus linearis* is the most common *Helophorus* we have collected at UMFS.

^d^ *Hydrocolus oblitus* is the most common small *Hydrocolus* at UMFS.

^e^ *Hydroporus rufilabris* represents >92% of *Hydroporus* we have collected at UMFS.

We combined beetles from both mesocosms to create a beetle assemblage, rather than only those that colonized the Add mesocosms. This aggregate assemblage is representative of beetles dispersing at the time of the experiment and hence would represent all taxa found in newly filled small lentic habitats in a landscape without established differences among ponds. Additionally, because our objective was to assess responses to beetles in general, combining beetles could help to build higher densities within these mesocosms in case of unforeseen effects beyond our control, such as mortality or spatiotemporal variability in dispersing populations. In turn, the densities of beetles added to Add mesocosms were approximately twice the density at which they naturally colonized mesocosms, but still well within the range of densities that can occur naturally (Fairchild et al., 2000, 2003; MRP *personal observation*). Additionally, mortality throughout the experiment (see results) likely meant densities were lower for most of the experiment. While beetles are prevalent across the landscape, habitat patches without beetles (our Remove mesocosms) do occur—seasonally such as when dry ponds refill or on shorter timescales such as when rainfall creates more ephemeral habitat patches.

### 2.2 End of the experiment

On 9 June, we measured the ammonium, temperature, specific conductance, dissolved oxygen, pH, and temperature of each mesocosm using a YSI Professional Plus meter to see if beetles potentially facilitated changes to abiotic characteristics of patches. We then collected zooplankton samples from mesocosms by collecting two 400 ml water samples from separate locations in each pool, filtering through 80 μm mesh into 50 ml centrifuge tubes, and preserving with Lugol's solution. We later counted and identified to order zooplankton in 1 ml subsamples from each 50 ml sample (Wetzel & Likens, 2000). We initially included total zooplankton abundance as a covariate in dytiscid analyses as higher zooplankton abundances may lead to higher colonization by dytiscids (Pintar & Resetarits, 2017a), but there was no effect here (*p* > .37), and we excluded this factor from all analyses. We terminated the experiment on 9 June and exhaustively collected beetles below the screens and sampled other insects by sweeping a fine mesh net around the pool until no debris remained. Only live beetles could be collected from below the screens as dead beetles typically break apart and are difficult or impossible to find among the leaf debris and identify.

Most beetles were identified to species (Tables 1 and 2). Other animals collected at the end of the experiment were identified to the following levels: Chironomidae larvae (non‐biting midges), Ephemeroptera larvae (mayflies), Anisoptera larvae (dragonflies), dytiscid larvae, *Berosus* larvae (Hydrophilidae), other hydrophilid larvae, and *Dolomedes* (fishing spiders).

**TABLE 2:** Species and abundances of small beetles (less than approximately 1.5 mm in width) that could potentially fit through gaps in screens covering mesocosms

Family	Identified level	Most likely species	Abundance		
			During	Analyzed	At end
Hydrophilidae	*Berosus exiguus*		6	6	0
Dytiscidae	*Bidessonotus inconspicuus*		4	4	1 (0 in Remove)
Hydrophilidae	*Derallus altus*		4	4	0
Dytiscidae	*Desmopachria*	*granum*	7	7	4 (3 in Remove)
Hydraenidae	*Hydraena marginicollis*		18	18	0
Dytiscidae	*Neobidessus pullus*		2	2	0
Hydrophilidae	*Paracymus*	*subcupreus* ^a^	289	289	6 (4 in Remove)
Dytiscidae	*Uvarus granarius*		37	37	0
Dytiscidae	*Uvarus lacustris*		52	52	0

Note

This table lists the species and their abundances as they were collected from above the screens every other day during the experiment (“During” column) as well as their abundances below the screens at the end of the experiment (“At end” column). None of these species were intentionally placed below the screens. The “Analyzed” column lists the number of individuals of those in the “During” column that colonized mesocosms after April and were included in analyses. We include the number of species collected below screens at the end of the experiment from Remove treatment mesocosms in parentheses in the “At end” column, with the remainder being in Add treatment mesocosms.

^a^ *Paracymus subcupreus* represents >95% of *Paracymus* we have collected at UMFS. These could also be *Crenitulus suturalis*, which are locally common at UMFS, but have not been collected from the location where this experiment was conducted.

### 2.3 Data analysis

We were unable to assess the effects of changes in beetle densities over time within mesocosms as we could not realistically track survival of each species throughout the experiment, which would require continuously destructively sampling below the screens. Therefore, cumulative sums of individual taxa (individual beetle species, frog eggs, mosquito egg rafts—see results) across the duration of the experiment served as our response variables of interest for most analyses, while categorical treatment (Add, Remove) was our predictor variable of interest. We did not expect equivalent responses among the various colonizing taxa, so we separately analyzed each abundant taxon. For all taxa with count data (abundances of eggs, egg rafts, or individuals), we constructed models in the same manner: We used linear mixed effects models fit by maximum likelihood using the Satterthwaite method with type III sums of squares to analyze the effect of treatment with pair nested within block as a random effect on square‐root transformed data using the lme4 package v 1.1‐23 and lmerTest package v 3.1‐2 in R v 4.0.2 (Bates et al., 2015; Kuznetsova et al., 2017; R Core Team, 2020). We include estimates of effect size ($\eta_{P}^{2}$) for our taxonomic response variables, and within each of three groups of taxa (beetles, ovipositing taxa, and other taxa at the end of the experiment), we include *P* values corrected for family‐wise error rates (Benjamini & Hochberg, 1995). However, the latter should be interpreted with caution as individual species are largely expected to be independent.

*Culex* was the only taxon with meaningful oviposition/colonization prior to establishment of treatments on 22 April. Therefore, we separately analyzed the cumulative number of *Culex* egg rafts oviposited in each treatment prior to beetle addition (Before group) and following beetle addition (After group). The Before group effectively serves as a baseline period during which there were no differences in treatments themselves, and therefore, we would not expect differences in *Culex* oviposition.

Frog oviposition began on 29 April, so we analyzed the cumulative abundance of frog eggs across the duration of the experiment. We individually analyzed the cumulative abundances of beetle taxa with abundances greater than 100, restricting our analysis to the colonists arriving after treatments had been established (24 April through 9 June). We also analyzed the abundances of zooplankton and other insect taxa with abundances greater than 100 collected at the end of the experiment with mixed effects models.

To assess overall effects on the beetle assemblage, we conducted three additional analyses. We assessed the (a) cumulative abundances of all colonizing adult beetles and (b) taxonomic richness of all beetles. The richness analysis included abundance as a covariate as the two are expected to positively covary. The (c) beetle assemblage structure was analyzed with PERMANOVA (adonis) to test for differences in multivariate centroid location (average community composition) between Add and Remove treatments with the vegan package v. 2.5‐6 (Oksanen et al., 2017). We analyzed the log‐transformed (except pH) environmental parameters of mesocosms with linear mixed effects models fit by maximum likelihood using the Satterthwaite method that included treatment as a fixed effect and pair nested within block as a random effect. The dissolved oxygen analysis included temperature as a fixed covariate.

## 3 RESULTS

**FIGURE 2:**
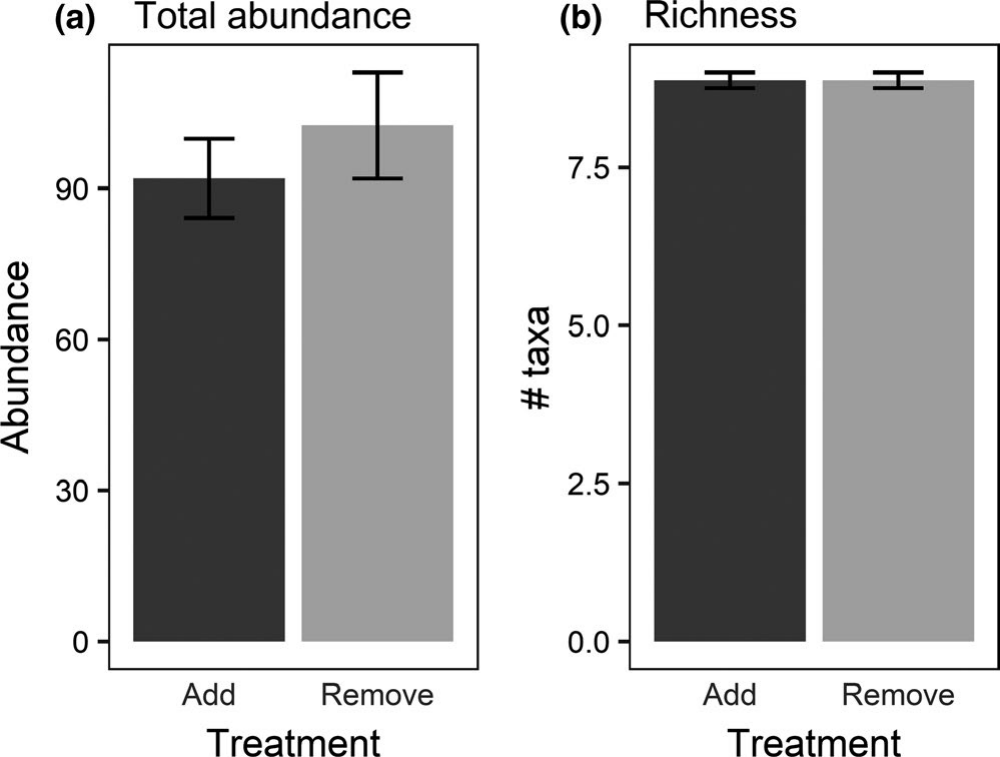
(a) Mean (± *SE*) total abundances per patch of all colonizing beetles in each treatment (Add = beetles added below screens; Remove = beetles removed). (b) Mean (± *SE*) per patch taxonomic richness of all beetles in each treatment. Data are the cumulative number of colonizing individuals that arrived from 24 April through 9 June

A total of 1622 beetles representing 40 taxa in 7 families colonized our experiment (Tables 1 and 2); 1165 of these beetles were placed below the screens of the pools (145.63 ± 13.46 beetles per Add mesocosm; mean ± SE), but only 344 were collected from below the screens at the end of the experiment (42.25 ± 9.41 beetles per Add mesocosm). There were no differences between treatments in the abundance of all beetles (*N* = 1556; Figure 2, Table 3a) or richness of beetles (*S* = 38; Figure 2, Table 3a). Analysis of the beetle assemblage showed there were no differences in assemblage structure between treatments (Table 3a).

**TABLE 3:** Analysis results for (a) beetle assemblage and individual taxa, (b) ovipositing taxa, (c) other taxa collected at the end of the experiment, and (d) environmental variables

	SS	*df*	*F*	*p*	*P* (BH)	$\eta_{P}^{2}$
(a) Beetles						
Beetle abundance	1.05	1, 8	1.11	.3228		0.080
Richness						
Abundance	0.45	1, 16	6.65	**.0202**		0.307
Treatment	0.01	1, 16	0.13	.7279		0.008
Assemblage structure						
Treatment	0.009	1, 11	1.11	.3677		0.092
Block	0.073	3, 11	3.11	.0001		0.459
*Berosus infuscatus*	0.074	1, 12	0.12	.7300	0.9091	0.009
*Copelatus glyphicus*	2.72	1, 8	8.83	**.0178**	**0.0367**	0.485
*Helophorus linearis*	1.76	1, 12	8.58	**.0126**	**0.0367**	0.408
*Paracymus*	2.66	1, 8	8.04	**.0220**	**0.0367**	0.424
*Tropisternus lateralis*	0.00	1, 12	0.01	.9091	0.9091	0.001
(b) Ovipositing species						
*Hyla chrysoscelis*	1551.3	1, 8	7.77	**.0237**	**0.0356**	0.414
*Culex restuans*						
Before	0.0094	1, 16	0.05	.8272	0.8272	0.003
After	6.7371	1, 8	138.19	**<.0001**	**<0.0001**	0.931
(c) Other taxa at end of experiment						
Chironomidae	3.05	1, 8	0.21	.6585	0.7911	0.013
Dytiscidae larvae	0.37	1, 12	0.10	.7518	0.7911	0.007
Ephemeroptera	0.58	1, 8	0.08	.7911	0.7911	0.007
Rotifera	7.09	1, 16	1.00	.3318	0.7911	0.059
(d) Environmental variables						
Ammonium	0.0011	1, 8	0.49	.5052		
Dissolved oxygen						
Temperature	0.0047	1, 16	3.09	.0980		
Treatment	0.0007	1, 13	0.42	.5270		
pH	0.0462	1, 8	2.11	.1840		
Temperature	0.0015	1, 8	20.96	**.0018**		
Specific conductance	0.0011	1, 8	0.24	.6396		

Note

Bold indicates statistical significance (*p* < .05); italics indicates marginal results (.05 < *p* < .10). All results are for the effect of treatment (Add versus Remove), except for richness, assemblage structure, and dissolved oxygen, which include multiple factors listed. *p* (BH) are *p* values adjusted for the false discovery rate via the Benjamini–Hochberg procedure (Benjamini & Hochberg, 1995). $\eta_{P}^{2}$ is an estimate of effect size.

**FIGURE 3:**
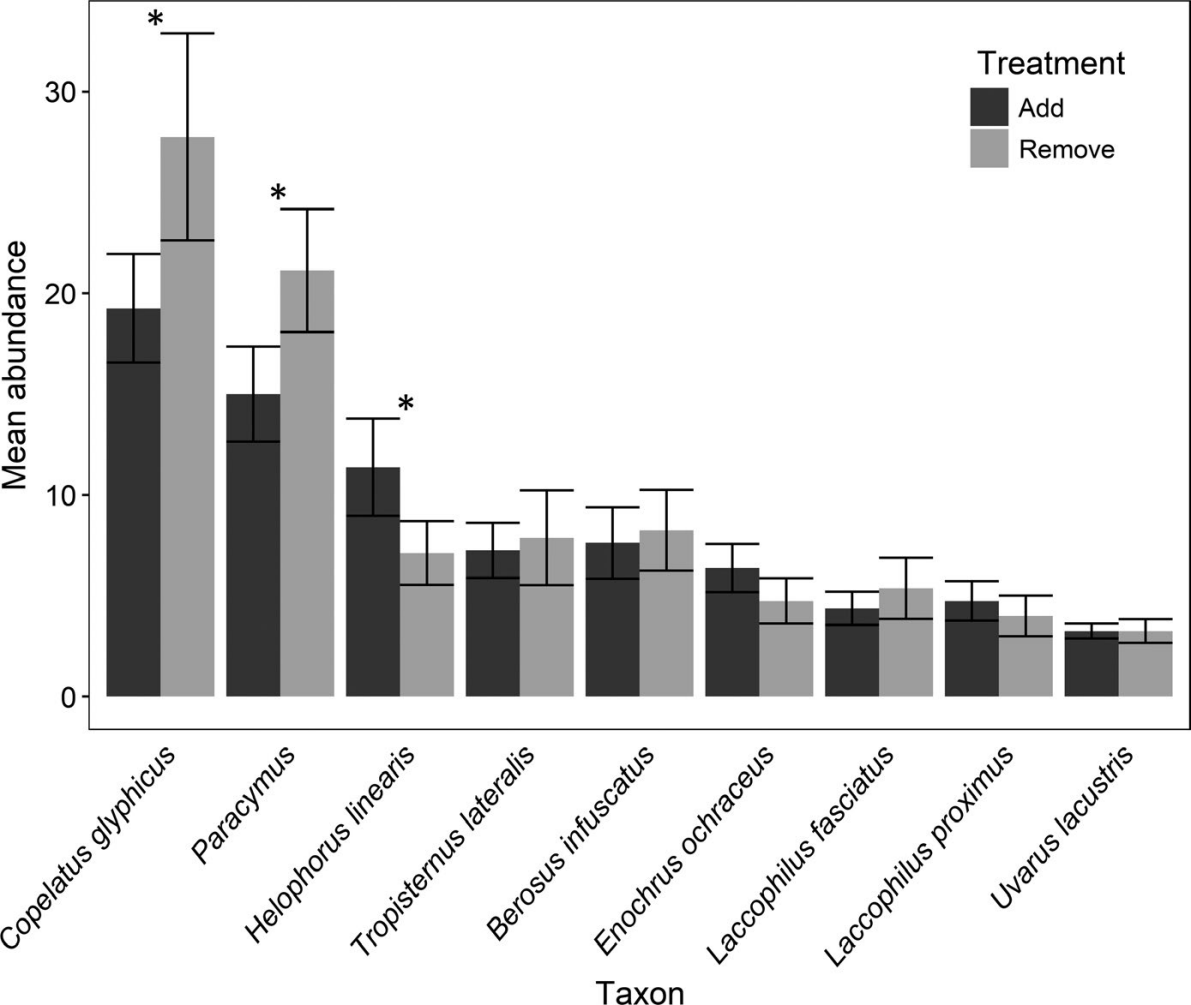
Mean per patch abundances (± *SE*) of colonizing beetles in each treatment (Add = beetles added below screens; Remove = beetles removed). Data are the cumulative number of colonizing individuals that arrived from 24 April through 9 June. We only analyzed the left five taxa with cumulative abundance > 100. The right four species (*N* > 50) are shown for illustrative purposes. Asterisks indicate significant differences in number of colonists between treatments (*p* < .05)

Five beetle taxa had total abundances >100 after treatments were established and were above our analysis threshold. Two beetle taxa colonized mesocosms that received beetles (Add) at lower rates than those that had beetles removed: *Copelatus glyphicus* (*N* = 376) and *Paracymus* (*N* = 289) (Figure 3). One beetle species, *Helophorus linearis* (*N* = 148), colonized mesocosms that received beetles at higher rates than those that had beetles removed. Two beetle species had no significant responses to treatment: *Berosus infuscatus* (*N* = 127) and *Tropisternus lateralis* (*N* = 121) (Figure 3).

**FIGURE 4:**
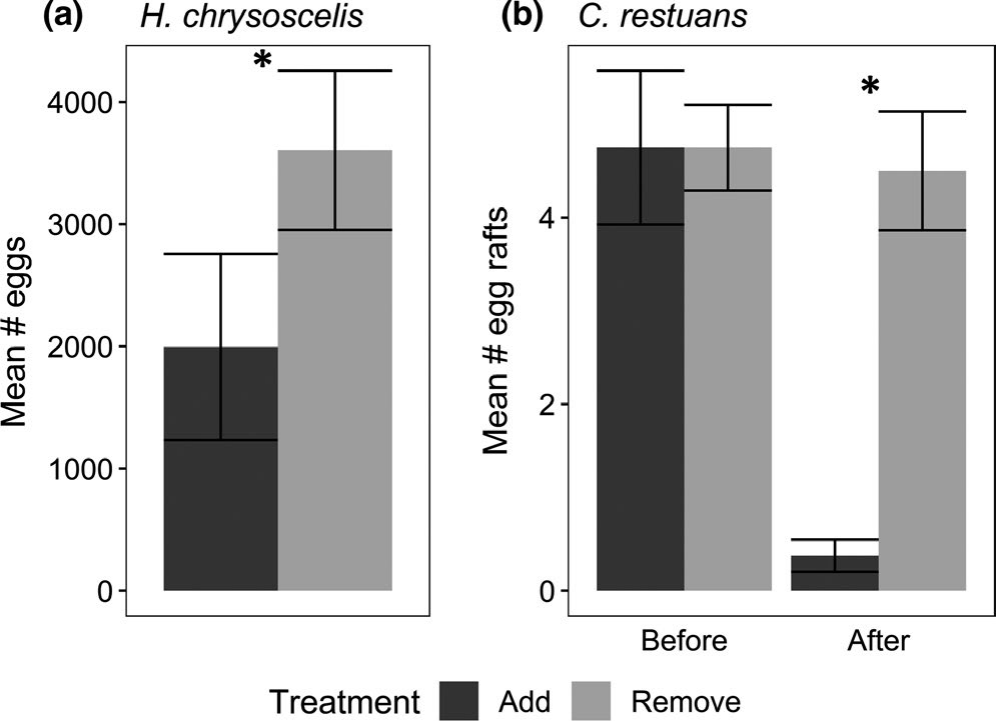
(a) Mean number of *H. chrysoscelis* eggs per patch (± SE) laid in each treatment across the duration of the experiment. (b) Mean per patch number of *C. restuans* egg rafts (± *SE*) oviposited in each treatment in the Before and After time periods. Asterisks indicate a significant difference between treatments (*p* < .05). Add = beetles added below screens; Remove = beetles removed

Two frog species oviposited in our experiment: *Hyla chrysoscelis* (Cope's gray treefrog) and *Gastrophryne carolinensis* (eastern narrowmouth toad). Oviposition by *G. carolinensis* was too limited to analyze (five mesocosms across two nights): They oviposited 941 total eggs in four mesocosms with beetles removed and 416 eggs in a single mesocosm with beetles added. Across 15 nights of oviposition, *H. chrysoscelis* oviposited significantly more eggs in mesocosms that had beetles removed (28,829 eggs) than those that had beetles added (15,961 eggs) (Figure 4a, Table 3b).

*Culex* mosquitoes oviposited a total of 115 egg rafts in our experiment from 18 April through 30 April. We continued searching for egg rafts for one week after 30 April, but none were observed. Prior to establishment of treatments on 22 April, 79 egg rafts were oviposited, and there were no differences between assigned treatment pools during this initial period (Table 3b). After establishment of treatments, 39 egg rafts were oviposited, with significantly more in mesocosms that did not have beetles added (Table 3b, Figure 4b). While we did not identify the larvae of any of our *Culex* egg rafts to species, across years, seasons, locations, and experiments at UMFS, >99% of several thousand egg rafts identified have been *Culex restuans* (Bohenek et al., 2017; unpublished data). Therefore, we assume that the 115 *Culex* egg rafts oviposited were *C. restuans*.

Of the taxa collected at the end of the experiment, we observed no differences between treatments in the abundances of chironomid larvae (*N* = 1069), Ephemeroptera nymphs (*N* = 1876), or dytiscid larvae (*N* = 450) (Table 3c). Abundances of Anisoptera nymphs (*N* = 54), *Berosus* larvae (*N* = 19), *Dolomedes* (*N* = 2), and other hydrophilid larvae (*N* = 1) were too low for analysis. Among zooplankton, rotifer (*N* = 469) abundance did not differ between treatments (Table 3c), while copepods (*N* = 97) were below our analysis threshold. No other zooplankton orders were found in samples.

The temperature of mesocosms with beetles added (19.34 ± 0.14°C; mean ± SE) was significantly higher than those that had beetles removed (18.94 ± 0.08°C) (Table 3d). Ammonium levels, specific conductance, and pH did not differ between treatments (Table 3d). Dissolved oxygen also did not vary between treatments, but had a marginal covariance with temperature: Dissolved oxygen trended lower when temperature was higher.

## 4 DISCUSSION

In temporary freshwater systems, the interplay between hydroperiod, predator–prey interactions, priority effects, and the many species that inhabit these systems creates landscapes of habitat patches that vary in numerous characteristics (Wellborn et al., 1996; Wilbur, 1997). Aquatic beetles are prevalent in these systems and can be some of the earliest taxa to arrive after ponds fill (Bilton, 2014; Fairchild et al., 2000), thus providing early variation in biotic characteristics among ponds within a landscape. Our results indicate the colonization and reproductive investment choices of a diverse group of animals (three out of five abundant beetle taxa, one mosquito, and one treefrog) using fishless freshwater habitats can be influenced by the presence of beetles. These habitat selection decisions should be made to maximize the expected fitness for each selective taxon (Chalfoun & Schmidt, 2012; Fretwell & Lucas, 1969; Morris, 2003).

While their natural history is generally poorly understood (Smetana, 1985), *Helophorus linearis* was the only common beetle species returned to pools that did not persist until the end of the experiment (Table 1). Most dispersal by adult *H. linearis* at UMFS appears to occur in a short window of time—less than a month—in spring (unpublished data; see also Landin, 1980). Thus, it may not be surprising that they selected patches containing other beetles (including conspecifics; Figure 3), perhaps showing conspecific attraction and facilitation (Sebastián‐González et al., 2010). If adults have a limited window in which to breed before they die, a primary factor influencing colonization would likely be the presence of conspecifics. Some *Helophorus* species are known to lose their flight ability after initial dispersal (Williams, 1996), placing greater emphasis on choosing a suitable habitat. If *H. linearis* selected for potential mates, conspecific presence was consistent across mesocosms with beetles added and may have been a reliable cue. Mortality of many of our beetles, and *H. linearis* in particular, was relatively high. Many of the species are likely short‐lived as adults, particularly smaller ones, although there are little species‐specific data on how long adults of our taxa survive. Survival (Table 1) tended to be higher among larger species, but it is possible screens on our mesocosms caused some mortality, though this did not prevent us from creating effective treatments.

In contrast to *H. linearis*, our two most common beetles, *C. glyphicus* and *Paracymus*, colonized mesocosms without added beetles at higher rates (Figure 3). *Paracymus* were not returned to mesocosms (placed under screens after initial colonization and collection), so intraspecific negative density dependence is obviated. We expect that the response by *Paracymus* is driven by avoidance of potential heterospecific competitors or predators. *Copelatus glyphicus*, a common temporary pond species (Miller & Bergsten, 2016), is the most common species that colonizes mesocosms at UMFS (Pintar & Resetarits, 2020), and it is responsive to variation in numerous patch characteristics, avoiding several predatory fish species (Resetarits & Pintar, 2016), selecting for patches with more nutrients and prey (Pintar et al., 2018; Pintar & Resetarits, 2017a, 2017d), and preferring smaller patches and those with closed canopy (Binckley & Resetarits, 2007; Resetarits et al., 2019). The relative importance of each of these factors remains largely unexplored, but at some point individuals are likely to exhaust options for available habitats and place lower importance on some factors that pose less immediate risk. Yet, for *C. glyphicus*, we still observed a moderate response here. The abundance and wide range of responses by *C. glyphicus* provide the possibility that this beetle could serve as a model species in habitat selection (Bilton et al., 2019).

The remaining two beetle species we analyzed, along with the patterns (unanalyzed) of the next four most abundant beetles (50 < *N* < 100), all showed no differences in colonization rates between treatments (Figure 3). Five of the six species do exhibit habitat selection in response to other factors, such as predator (fish) presence, patch size, and resource availability (Pintar et al., 2018; Pintar & Resetarits, 2017a, 2017b; Resetarits & Pintar, 2016). The exception is *U. lacustris*, which has not been common enough in other studies to analyze, though closely related *Uvarus granarius* does respond to predators (Resetarits & Pintar, 2016). Of the six remaining beetle taxa, their abundances could have been too low to illustrate meaningful patterns, overall densities of beetles in our pools were too low to generate a response, or they simply do not select patches based on the presence of either conspecifics or heterospecifics. Overall, these insect colonization patterns resulted in no differences in the structure of the adult insect assemblages arriving into patches. The lack of pattern in composite variables and assemblage analyses may not be reflective of what happens at the species level, given the species‐specific responses observed.

Species‐specific patterns (including nonresponses) like those we observed here are often observed in colonizing aquatic beetles across differences in numerous patch characteristics (Kraus & Vonesh, 2010; Pintar et al., 2018; Pintar & Resetarits, 2017a; Resetarits & Pintar, 2016; Turner et al., 2020). Integration of this variation in responses to varying patch characteristics has the potential to create considerable niche differentiation (Maire et al., 2012) at the colonization stage of the aquatic beetle lifecycle (Resetarits et al., 2019). However, the relative importance of the various patch characteristics remains largely unknown. In limited studies assessing multiple characteristics, predation risk appears to outweigh other factors, at least for most species (Pintar et al., 2018; Resetarits et al., 2019). This is not surprising as there is no better way to reduce future fitness than through death, and predators often have strong, lethal direct effects (Lima & Dill, 1990; Matassa & Trussell, 2011). Any direct lethal predatory effects in our experiment would have been limited to predation by adult beetles on the egg or larval offspring of colonists. Predation by adult aquatic beetles on other adult aquatic beetles (Culler et al., 2014) is something we do not observe in our experiments, with two exceptions: On a few occasions, we have observed adults of the largest dytiscids (*Cybister* and *Dytiscus*) kill other adult beetles, but both of these genera are rare in our mesocosms and were absent from this experiment. More commonly, adult *Notonecta* kill adult beetles of many species (M. R. Pintar and W. J. Resetarits, *in review*), but only three *Notonecta* colonized this experiment and none were placed below the screens (*Notonecta* strongly prefer larger patches; Resetarits et al., 2019). As adults, many of our beetle taxa would be expected to prey on organisms with more vulnerable morphologies than other adult beetles, such as larval insects or zooplankton (Culler et al., 2014; Herwig & Schindler, 1996).

The species‐specific nature of predation and competition among aquatic beetles is relatively poorly understood considering the high diversity of species (Vamosi & Wohlfahrt, 2014). However, we expect that reduced colonization is not a response to predation risk on adults, but to competition among adults, competition among offspring, and/or predation on offspring (Culler et al., 2014). How, or if, colonizing adult beetles weigh the importance of various factors to themselves and their offspring is unknown. While we did not directly measure beetle oviposition (which for most species is not as remotely tractable as for frogs or *Culex*), we would expect a positive correlation between oviposition rate and adult colonization rate because many breed soon after initial colonization (Resetarits, 2001).

In our direct measurements of *H. chrysoscelis* oviposition, they selected sites based on the presence of beetles that could be predators or competitors of their offspring, depositing fewer eggs in mesocosms containing beetle assemblages than those without (Figure 4a). *Hyla chrysoscelis* also avoid ovipositing in habitats containing a wide range of vertebrate predators and competitors (Resetarits & Binckley, 2013; Resetarits & Wilbur, 1989), while they also have better survival and performance in recently filled ponds, and choose newly filled ponds over older ponds (Pintar & Resetarits, 2017c, 2017e; Wilbur & Alford, 1985). Our data now indicate that *H. chrysoscelis* can limit risk by responding directly to the presence of beetle assemblages. *Hyla* oviposition responses to other invertebrates, as well as any effects of hydrophilids on anurans, are not known. Larval and adult dytiscids are effective predators of *H. chrysoscelis* and other anuran eggs and larvae (Cronin & Travis, 1986; Formanowicz & Brodie, 1982; Gould et al., 2019; Resetarits, 1998; Roth & Jackson, 1987). We would expect larval beetles of both families, adult dytiscids, and potentially adult hydrophilids, to place some predation pressure on treefrog eggs and small early larval stages, but that only larger dytiscids (adults and larvae) would be effective predators of larger, late stage larval *Hyla*. Adult hydrophilids and other less common scavenging and herbivorous beetle families may compete with larval anurans, but competitive pressure should be less important than predation pressure (Lawler & Morin, 1993).

Similar to *H. chrysoscelis*, ovipositing *Culex* preferred mesocosms without beetles once treatments were established (Figure 4b). *Culex restuans* at UMFS are highly responsive to many species of predators, including numerous fish species, *Ambystoma* larvae, and two large dytiscid species, *Cybister fimbriolatus* and *Thermonectus nigrofasciatus* (*unpublished data*), but not the predaceous hemipteran *Notonecta irrorata* (M. R. Pintar and W. J. Resetarits, *in review*). Mosquitoes, and *Culex* in particular, have highly sensitive olfactory systems and are highly selective to a wide range of patch characteristics when choosing oviposition sites (Carey & Carlson, 2011; Kiflawi et al., 2002; Silberbush & Blaustein, 2011; Vonesh & Blaustein, 2010), enabling them to effectively avoid predators. Two of our abundant beetle species, *L. fasciatus* and *L. proximus*, are documented predators of mosquito larvae (Bofill & Yee, 2019; Pitcher & Yee, 2014), so we expect dytiscids similar in size to *Laccophilus* (e.g., *C. glyphicus*), and those larger, to also be effective predators of *Culex* larvae (Batzer & Wissinger, 1996; Larson et al., 2000). *Culex* larvae are highly vulnerable to predation by many predator taxa, which may be an added benefit to oviposit in newly filled pools. Early oviposition by *Culex* should result in higher per capita resource availability due to lower competitor abundance and higher survival due to lower predator abundance (Chandrasegaran & Juliano, 2019; Ower & Juliano, 2019).

Due to the very nature of our system, we are unable to elucidate responses by colonists to specific species of beetles present in our assemblages. While chemical cues predominate in the assessment of patches based on the presence of animals (Eveland et al., 2016), the identity of these chemical cues is not known for almost all species (but see Silberbush et al., 2010 and Landeira‐Dabarca et al., 2019). It is also unknown whether individual aquatic beetle species produce unique chemical cues. If all beetles produce the same or a very similar set of perceived chemical cues, the colonization responses should be the same regardless of which set of species were present. This seems somewhat unlikely, however, given that mating in insects often involves species‐specific pheromones and/or receptors (Nakagawa et al., 2005; West‐Eberhard, 1984), and heterospecifics may eavesdrop on such cues (Stowe et al., 1995; Symonds & Elgar, 2008). In addition, we have observed variation in colonization responses to a wide range of fish species and interactions to combinations of fish species that can be unpredictable based on responses to individual species (Resetarits & Pintar, 2016) (*unpublished data*). Nevertheless, how colonists respond to individual species remains an interesting question with abundant opportunity for future research.

Environmental parameters of our mesocosms generally suggested that our movement of beetles had no effect on environmental conditions, with the exception of higher temperatures in mesocosms with beetles added. Higher temperatures perhaps could be due to greater mixing of the water column via beetle movement, but this movement effect would likely be minimal relative to the act of collecting beetles with nets every other day in the shallow mesocosms, and experimental evidence for it is lacking. Conversely, the added beetles could have led to greater processing of leaf litter and bioturbation in the mesocosms (Adámek & Maršálek, 2013), perhaps resulting in darker coloration of the water, enabling greater heat absorption. However, if there had been greater leaf litter breakdown, we might also expect increased conductivity due to more material in the water column, but conductivity did not differ between treatments. By us moving insects in our methods, we could transport some nutrients between patches and increase chlorophyll production, but ammonium levels did not differ between treatments and we did not measure chlorophyll, although there were no apparent visual differences between mesocosms. Although the reason for these higher temperatures remains undetermined, some aquatic beetles select patches with cooler temperatures (McNamara et al. 2020), however the difference in temperature they reported (~4.5°C) was much larger than we did here (0.4°C). Hence, we do not expect such a small temperature difference would generate the larger effect sizes and variation in colonization patterns we observed, which are more characteristics of responses to predation risk (Resetarits & Pintar, 2016; Resetarits et al., 2019).

The response of our most common taxa to the presence of beetle assemblages places further emphasis on the benefits gained when individuals are among the earliest arrivals at ponds after filling (Pintar & Resetarits, 2017c; Wilbur & Alford, 1985). Dispersal and colonization are important processes that connect local populations and communities into metapopulations and metacommunities (Leibold et al., 2004; Resetarits & Silberbush, 2016; Trekels & Vanschoenwinkel, 2019), and that importance is quite clear in temporary freshwater systems. Because patterns of dispersal, colonization, and other aspects of the phenology of animals in temporary pond systems can be very cyclical and episodic, habitat selection for ponds without existing beetle assemblages might generate greater spatiotemporal niche separation among the large number of species using temporary ponds. Habitat selection plays an important role in the patterns of colonization and resulting community structure in habitat patches and across landscapes (Kraus & Vonesh, 2010; Resetarits & Pintar, 2016; Resetarits et al., 2019; Vonesh et al., 2009). Determining and integrating how species interact at both the colonization stage, and through post‐colonization processes such as predation and competition, are vital to understanding the patterns of species abundance and diversity observed in natural landscapes.

## CONFLICTS OF INTEREST

The authors declare no conflicts of interest.

## AUTHOR CONTRIBUTION

**Matthew R. Pintar:** Conceptualization (lead); Data curation (lead); Formal analysis (lead); Investigation (lead); Methodology (lead); Visualization (lead); Writing‐original draft (lead); Writing‐review & editing (lead). **William J. Resetarits:** Formal analysis (supporting); Funding acquisition (lead); Resources (lead); Writing‐review & editing (supporting).

### Open Research Badges

This article has earned an Open Data Badge for making publicly available the digitally‐shareable data necessary to reproduce the reported results. The data is available at https://doi.org/10.5061/dryad.qz612jmcj.

## Data Availability

Data are available in Dryad at https://doi.org/10.5061/dryad.qz612jmcj.
